# Unlocking the Potential of Vitamin D: A Comprehensive Exploration of Its Role in Neurological Health and Diseases

**DOI:** 10.3390/biology14030280

**Published:** 2025-03-10

**Authors:** Rehana Khatoon

**Affiliations:** Department of Medical Elementology and Toxicology, School of Chemical and Life Sciences, Jamia Hamdard, New Delhi 110062, India; rehanakhatoon_sch@jamiahamdard.ac.in; Tel.: +91-11-26059688 (ext.5573); Fax: +91-11-26059663

**Keywords:** vitamin D, oxidative stress, inflammation, cell death, neurological disorder

## Abstract

Vitamin D (VD), an indispensable micronutrient renowned for its pivotal role in bone health, is increasingly recognized as a frontline therapy for bone-related disorders owing to its involvement in maintaining calcium/phosphorus levels. Beyond these benefits, VD exhibits a modulatory impact on redox imbalance, inflammation, and anti-apoptotic pathways implicated in brain-related disorders. Recent findings reveal a notable decrease in VD and its receptor expression in the cerebrospinal fluid of individuals with brain diseases, indicating a positive association between VD levels and normal brain function. Moreover, emerging reports underscore VD’s potential in mitigating the pathophysiology of neurodegenerative diseases, including memory and motor impairments, mitochondrial dysfunction, and neuronal loss. Extensive in vitro and in vivo studies elucidate VD’s multifaceted neuroprotective mechanisms, effectively mitigating neuronal damage and ATP deprivation, thus reducing mortality and morbidity. This review comprehensively examines VD’s diverse attributes, encompassing antioxidative, anti-inflammatory, anti-apoptotic, and neurogenic effects. It provides contemporary insights into VD’s efficacious actions at appropriate doses and exposures across diverse neurological experimental models. Furthermore, the clinical relevance of VD in treating patients with neurological diseases is explored. Overall, this review contributes to the exploration of potential neuroprotective agents and holds promise for improving human health outcomes in the future.

## Simple Summary

This review investigates the extended role of vitamin D beyond its traditional association with bone health, focusing on its influence on brain-related disorders. It highlights vitamin D’s involvement in regulating oxidative stress, inflammation, and anti-apoptotic pathways within the brain, with lower levels being linked to several neurological diseases. Vitamin D shows promise in reducing the risk or progression of neurodegenerative conditions through various protective mechanisms. This review added evidence from in vitro, in vivo, and clinical studies that demonstrate the positive effects of vitamin D across a range of different neurological disorders. In conclusion, vitamin D emerges as a potential neuroprotective agent with considerable relevance to human health.

## 1. Introduction

Vitamin D (VD), an essential steroid of our body, is crucial for maintaining the calcium and phosphorus levels for healthy bones [1]. In addition to this activity, VD still needs an immense investigation due to its involvement in the regulation of other essential biological and physiological machinery of different organs in the body. According to studies, the brain, in particular, expresses high levels of VD receptors and enzymes in different regions, which is necessary for its metabolism, indicating its importance in the neurodevelopment process and essential functions [2]. Many researchers have established correlations between VD levels and various brain-related pathologies, including motor dysfunction, dementia, and anxiety, with alterations in specific brain regions contributing to these conditions [3,4]. Similarly, a study confirmed the inverse relationship between VD levels and the incidence of dementia and motor dysfunction, highlighting the potential implications of VD deficiency in the aging population [5,6]. A recent study has shown significant concentrations of VD metabolites in different brain regions, like the hippocampus and substantia nigra, play a prominent role in the regulation of memory formation and motor coordination [7]. Furthermore, a study confirmed that VD supplementation has been demonstrated to enhance memory processes and mitigate motor function impairments. Anxiety, particularly prevalent in those with reduced VD levels in the prefrontal cortex, further illustrates VD’s multifaceted role in mental health [8]. Body weight changes in VD-deficient individuals also raised the possibility of VD involvement in hypothalamic function regulation [9]. According to a study by Zhu et al., 2018, it was shown that VD supplementation for 6 months in VD-deficient patients reduced anxiety but not depression symptoms [10].

Hence, a number of studies are available that confirm VD’s impact on the onset and development of symptoms in brain-related disorders like Parkinson’s, Alzheimer’s, Huntington’s, etc [11,12,13]. Not surprisingly, a number of available recent clinical and preclinical reports also documented the association of VD deficiency severity with the onset of different neurological illness symptoms [14]. In this regard, the purpose of this review is to summarize the current information on VD’s role as an effective therapeutic invention for the regulation of prominent causative mechanisms management and increasing the understanding of VD use for curing different neurological disorders at preclinical and clinical levels.

## 2. Metabolism of VD

**Figure 1:**
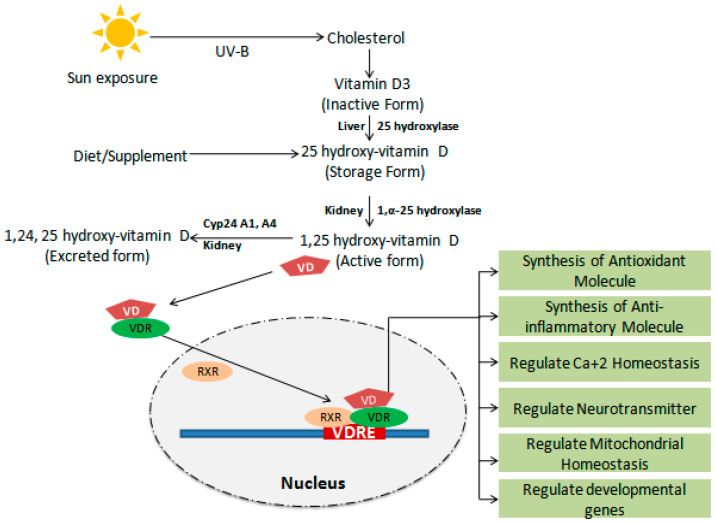
VD metabolism and functions: In the skin cells, UV-B light exposure from the sun induces the conversion of cholesterol to pro-vitamin D forms. Pro-vitamin D enters the liver and is converted into 25 hydroxy-vitamin 3 (cholecalciferol) via the 25-hydroxylase enzyme. This is the first hydroxylation step of active form vitamin D synthesis, and it is also taken from external food sources. The second step of hydroxylation is initiated in the kidney by 1,α-25 hydroxylase enzyme and converted into 1,25 hydroxy-vitamin D, which is an active form of vitamin D. The active form binds with the vitamin D receptor (VDR), enters the nucleus, and regulates the synthesis of antioxidant, anti-inflammatory molecule, Ca^+2^ homeostasis, neurotransmitter, mitochondrial, and developmental genes via the relevant specific gene transcription machinery activation. The active form of VD is regulated by kidney resident Cyp24A1 and A4 enzyme by feedback mechanism activation.

Knowledge of VD metabolism is very important to better understand the mechanism of action (Figure 1). VD is a fat-soluble sterol occurring in ergocalciferol (VD2) and cholecalciferol (VD3) forms. VD2 is essentially found in plants, and VD3 is formed in the human skin by a photochemical reaction using ultraviolet B light [15]. For the active form, it undergoes two enzymatic hydroxylation reactions. Firstly, it is transported to the liver with the help of a carrier protein, and here, it is hydroxylated into 25(OH) D or calcitriol by the vitamin D-25-hydroxylase (CYP2R) enzyme. Calcitriol is again transported in the kidney for the second hydroxylation step and converted into the biologically active form 1, 25-dihydroxyvitamin D (1, 25(OH) 2D) or calcitriol by 25(OH) D-1-OHase alpha hydroxylase enzyme (CYP27B1). Recent evidence suggests that the kidney is not the only organ where the second-step hydroxylation occurs. The enzyme involved in hydroxylation is also present in keratinocytes, monocytes, macrophages, osteoblasts, prostate, and colon cells [15,16]. Recently, the presence of CYP27B1 enzyme has also been confirmed in the brain. The circulating 25(OH) vitamin D form easily enters neuronal and glial cells by crossing the blood–brain barrier and is converted to the biologically active 1,25-dihydroxyvitamin D (1, 25 (OH) 2D) form. Another study confirms that the level of active form of VD in the brain is maintained by another catabolizing enzyme, Cytochrome P450 family 24 subfamily A member 1 (CYP24A1). CYP24A1 is involved in the conversion of 1, 25 (OH) 2D into the inactive calcitroic acid form [16]. Evidence suggests that mRNA expression of CYP24A1 has been increased with 1, 25 OH VD level change in the glial cells [17,18]. Thus, culminating the above clues reveals that 1, 25(OH) 2D is formed in multiple organs, including the brain, for specific functions and is regulated in an autocrine manner.

## 3. Action Mechanism of VD

**Figure 2:**
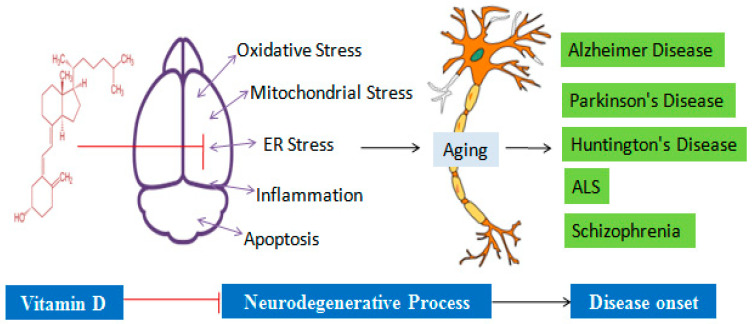
VD-mediated regulation of causative factors in different neurological conditions. Oxidative stress, mitochondrial dysfunction, ER stress, inflammation, and apoptosis in neuronal cells unleash the aging process as a consequence of the onset of Alzheimer’s, Parkinson’s, Huntington’s, ALS, and schizophrenia diseases. VD normalizes the process of these causative agent mechanics and reduces the chances of disease onset and pathogenesis in the brain.

**Figure 3:**
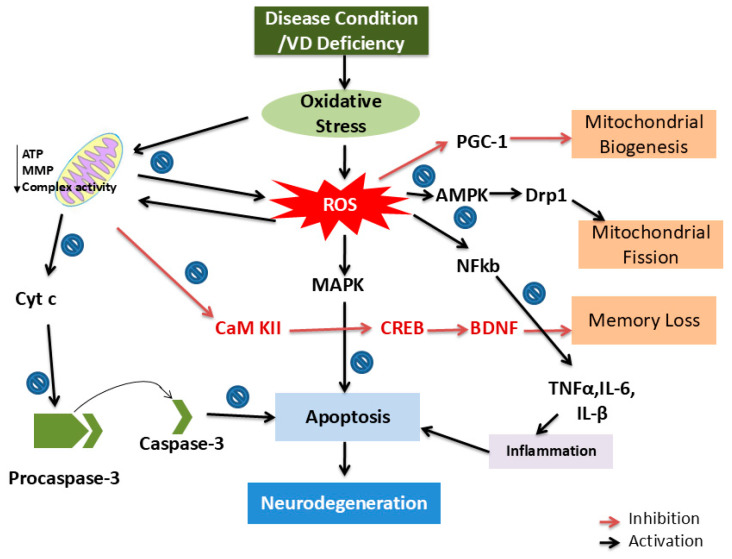
Probable mechanisms involved in the onset and pathogenesis of different neurological diseases and their regulation by VD: In neurological disease conditions, VD deficiency occurs and induces oxidative stress, which is ultimately linked to mitochondrial dysfunction. Mitochondrial dysfunction is the cause of ATP depletion, reduced mitochondrial complex activity, and mitochondrial membrane permeability disruption. As a result of the disruption of membrane permeability, this decreases the affinity of cyt c with the inner mitochondrial membrane and promotes the release of cyt c in the cytosol from mitochondria. Cyt c induces the apoptosis process protein via the cleavage of pro-caspase-3 to caspase-3, and the loss of neuronal cells occurrs. Mitochondria also regulate the balance of Ca^+2^ in the cytosol and mitochondria, and due to impairment in the normal function of mitochondrial activities, this decreases the CaMKII/CREB/BDNF pathway, and eventually, the event of memory loss is initiated. In addition, oxidative stress involved in ROS formation by the mitochondrial respiration alteration or other components of the body, like NADPH oxidase, regulate mitochondrial biogenesis and mitochondrial fission through the AMPK and PGC-1 pathways. ROS induce inflammation via the activation of the NFkB pathway, which is also with the apoptosis of neuronal cells. On the other hand, the induction of the MAPK pathway by excessive ROS generation also participated in the neurodegeneration process by apoptosis induction. Abbreviation: TNFα = tumor necrosis factor, IL6 = interleukin-6, Cyt c = cytochrome c, CREB = cAMP-response element binding protein, CaMKII = calcium/calmodulin-dependent protein kinase II, PGC1 = peroxisome proliferator-activated receptor-gamma coactivator 1, Drp1 = dynamin-related protein 1, ATP = adenosine triphosphate.

VD action is exerted by the nuclear or plasma membrane VD receptor (VDR). As with the VD hydroxylation enzyme, the gene encoding the receptor for VD is present in many tissues and functionally involved in 3% of human genome regulation. The presence of VDR mRNA expression has been confirmed in different regions of the brain, including the hippocampus, cerebellum, astrocyte, and oligodendrocyte [19]. The importance of VD in the regulation of different aspects of brain development, differentiation, and cellular machinery is significantly defined by its expression presence [20]. It behaves like a hormone and performs genomic and non-genomic functions. The receptor belongs to the nuclear family with DNA binding domains and is involved in the genomic action of VD. After binding, it is translocated into the nucleus and binds with the VD response element (VDRE) in the promoter region, leading to the transcription regulation of certain genes that are involved in the neurogenesis and survival of the neurons (Figure 2 and Figure 3). On the other hand, in non-genomic action, VD is involved in the regulation of redox imbalance, release of inflammatory molecules, and inhibition of apoptosis in different neurological conditions [2]. The different aspects of VD are discussed in later sections.

### 3.1. VD and Oxidative Stress

An imbalance of redox in the cells is a situation where the generation of reactive oxygen species (ROS) is enhanced in relation to the antioxidant present. A recommended level of ROS is mandatory inside the cells because of its role in the regulation of proliferation, differentiation, and survival processes, but its generation beyond the level promotes the necrosis and death mechanism of the cells. Increased ROS levels promote oxidative damage through the oxidation of proteins, lipids, and DNA molecules [21,22]. The concluding remarks of the available studies have evidenced that enhanced levels of oxidized proteins and lipids because of imbalanced redox play a crucial role and are considered an early event in various neurogenerative diseases [23]. From this point of view, the antioxidant potency of VD has drawn keen attention for the management of redox imbalance. A much-debated question is whether VD acts directly on the disposition of free radicals or indirectly by activating essential pathways for neuroprotection. This area is not fully studied in the pathologies of different neuronal disease conditions. The proof of a direct impact rests on the fact that VD hampers the production of ROS and prevents their hydroxyl donor [24]. A study confirms that the effect of VD on ROS and OH has been increased with the presence of progesterone steroid hormone [25]. VD also affects metal-induced Fenton reactions and ROS formation. VD pretreatment significantly reduced lipid peroxidation and apoptotic protein release in iron-treated locus coeruleus of rat brains [26]. Iron-related oxidation and accumulation of ferric iron in the neuronal cells are also prevented by VD administration [27]. In a similar way, VD also reduced ROS formation and cell death in zinc-exposed cortical neuronal cells [28]. On the other hand, the indirect impact of VD on oxidants is also studied. A study suggests that VD promotes the survival of the cells by enhancing sodium dismutase, glutathione peroxidase, and catalase antioxidant enzyme activity in the hippocampus region of rat brains [29]. Nrf2, a prominent, well-known transcription factor for antioxidant enzyme synthesis, has been enhanced by VD treatment in the striatal region of rat brains [30]. Thus, the above evidence cumulatively proves the efficacy of VD as an antioxidant in different neurological disease conditions.

### 3.2. VD and Inflammation

Inflammation in the neuronal cells is believed to be a key driving force in the onset and progression of neurodegenerative diseases. It is triggered by the over-activation of innate immune response cells, including microglia or astrocytes, in response to protein misfolding or other environmental stress and diverts it from the beneficiary function to the sustained release of pro-inflammatory molecules [21]. In addition to the antioxidative effect, a recent study also confirmed that VD has the potency to slow down the inflammatory storm that ultimately leads to reduced neuronal loss [31]. VD decreased the activation of NFkB transcription factor and related protein interleukin-6 (IL-6), IL-1β, and tumor necrosis factor (TNF-α) in the different cell types, suggesting the neuroprotective mechanism [32]. Interestingly, in transgenic 5XFAD Alzheimer’s mice, VD reduced the marker of activated microglia and iba-1 and TNF-α protein in the cortex and hippocampal regions of the brain [33]. Reduced mRNA expression of IL-6, IL-1β, and TNF-α and p62 were also reported in the LPS-induced inflammatory rat models [11]. Moreover, a recent study suggested that modulation of the inflammasome/caspase-1 pathway also plays a significant role in inflammation-induced neuropathogenesis. A study confirmed that inflammasome signaling regulating NLR family pyrin domain containing 3 (NLRP3) protein interacts negatively with VDR. VD supplementation inhibits the oligomerization of NLRP3 with Lys-36-specific deubiquitinase (BRCC3) protein for activation and optimizes the signaling toward the normal range in the LPS-induced model [34]. Still, more studies are required to explore the mechanism, but it appears to have a hugely affirmative action on inflammation. Collectively, evidence confirmed that neuroinflammation is a very well-known mechanism related to neurodegeneration, and thus, VD could be used as a valuable tool and therapy in this area for cure.

### 3.3. VD and Apoptosis

The mechanism of apoptosis is an empirical event that occurs in most neurodegenerative diseases. Two types of pathways of the apoptosis mechanism are studied in stress conditions. The first is the mitochondria-mediated pathway, known as the intrinsic pathway, and the other one is a receptor-mediated pathway, known as the extrinsic pathway [35]. The mitochondrial-mediated pathway is mostly studied in neurological disease conditions. Nevertheless, the information about the extrinsic apoptotic pathway is comparatively limited. In the intrinsic pathway, various proteins, such as Bax, Bcl2, cytochrome c, and caspase-3, are participants [36]. In this regard, understanding the anti-apoptotic action of VD will be of keen interest, as it already confirms the number of disease conditions. A recent study suggests that VD3 treatment in apoptotic neurons mitigates the enhanced proapoptotic factor Bax level and the activity of caspase-3 [37]. In hypoxic H_2_C_9_ cells, VD significantly reduced TUNEL-positive neuron cell number [38]. VD administration also inhibits impaired neuromuscular coordination, neuronal death, and DNA damage in the hippocampus and serum of Alzheimer’s rat models [12]. It attenuates apoptosis and oxidative stress in neurons and mesenchymal stem cells by upregulating the anti-apoptotic protein Bcl_2_ [39]. It inhibits the ethanol-induced increased ROS level and increases Bcl-2 expression in murine HN9.10e hippocampus-derived cell lines and animal models [40]. VD showed potency in regulating the alteration in mitochondrial function, including mitochondrial membrane potential, electron transport chain deficiency, ATP synthesis, and decreased antioxidant enzymes, as well as halted the translocation of cyt c and activation of caspase in the Parkinson’s and ataxia rat models [41,42]. VD significantly inhibits the swelling in mitochondria and increases VDAC, hsp60, and caspase-3 expressions and cell death in 6-OHDA-administered rats [43]. Caspase-3-mediated cell death in cerebral ischemia is also inhibited by VD treatment [44]. VD is essential for mitochondrial structural integrity, Ca^+2^ homeostasis, and neuritic regeneration in the dorsal ganglial neurons of ataxia disease models [43]. LPS-induced mitochondrial ROS level, activation of the c-Jun N-terminal kinases (JNK) pathway, cleavage of caspase-3, and death in cortical and glial primary culture cells were mitigated by VD treatment [45]. A number of studies are available to confirm the anti-apoptotic potency of VD in cell culture and rodent model studies. It reduces the generation of β-amyloid and synuclein aggregation in the brain region of AD and PD models of rats, respectively, by showing the nature of anti-apoptosis [12,41]. Though several reports have shown the anti-apoptotic effect of VD on the mitochondria-mediated apoptotic pathway, studies are still required to investigate its effect on the extrinsic apoptotic pathway [46]. In addition, autophagy is also one of the cell death processes occurring in the progression of pathogenesis of neurological diseases, and such mechanisms are genetically governed and conserved evolutionarily to regulate the fate of cells. The process of autophagy is intimately interconnected with the apoptotic mechanism by the regulation of some apoptotic proteins, such as Bax and Bcl2 [47]. Another study has shown that VD downregulates autophagy by increasing the expression of Bcl_2_ and Bcl_-xL_ proteins in the cortical region of ischemic brains [38]. VD significantly reduces the LCII/LC1 ratio and restores autophagy influx with the help of the phosphoinositide 3-kinases (PI3K)/AKT pathway in the cortical region of the traumatic brain injury model of rats [48]. Thus, the above finding confirmed the prominent role of VD in apoptosis regulation and opens the door to the search for effective drugs to halt the process of neuronal cell death in neurological diseases.

### 3.4. VD and Neurotrophins

Neurotrophins (NGF, GDNF, NT1, NT2), essentially expressed in the brain cells, are growth factors and are involved in the growth, development, neurotransmission, and synaptic plasticity of neurons. Because of their versatile function in normal brain function, alterations in the expression of different neurotrophins have been studied in neurological disease conditions and are correlated with the degeneration of neurons. Recently, studies have confirmed VD involvement in neurotrophin expression at gene and protein level and function. In a study, VDR silencing in the primary cortical neuron caused a significant decrease in nerve growth factor (NGF) expression [49]. Another study suggests that VD supplement in the hippocampal region of rat brains significantly enhances the production of NGF [50]. PI3K/tropomyosin receptor kinase B (Trkb) signaling is a major regulator of neurogenesis and NGF expression. In the HD mice, VD-mediated enhancement of Trkb, PI3K, and NGF expression has been reported. In PC12 cells, htt gene expression has been modulated by the NGF, and its reduction increases htt expression, which suggests the possible neuroprotective mechanism of VD in HD. GDNF, an essential component of dopaminergic neural differentiation and survival, has also been linked to VD because of broader trophic action in developing adult brains. A study confirmed that VD significantly enhances the growth-derived nerve factor (GDNF), brain-derived nerve factor (BDNF), and neurotrophin-type 3 (NT3) expression in neuronal stem cells [51]. Cultured mesencephalic neuronal cells, prominently used for the dopaminergic neuron studies, showed a significant increase in GDNF expression with dopamine neuron (DA) numbers after VD treatment [52]. In addition to NGF modulation, VD-mediated enhancement in the expressions of neurotrophin-3 and -4 has also been confirmed in neuroblastoma cells. Thus, the above information advocates for the beneficial effect of VD on neurotrophin function modulation and suggests a way to be used as an effective agent in brain-related alterations.

## 4. VD Treatment in Neurodegenerative Diseases

The term neurodegeneration is related to the loss of neurons participating in motor, sensory, and cognitive functions. The selective neuronal cell vulnerability is one of the most notable features of every neurodegenerative disease, including Alzheimer’s disease, Parkinson’s disease, Huntington’s disease, amyotrophic lateral sclerosis, and schizophrenia. The main etiology behind each neurodegenerative disease is the presence of oxidative stress, inflammation, apoptosis, and reduction in neurotrophin levels in the brain [20,53]. These hypotheses are considered in view of being able to account for cumulative complications linked with the onset and progressive nature of these disorders. Understanding the extent and the link among these factors may lead to the discovery of effective pharmacological interventions that interfere with such a series of pathological events (Figure 2 and Figure 3). In this context, VD grabbed the researchers’ attention to look into its remedial impact on neurodegenerative diseases (Table 1). In this section, we include information about the effect of VD on different neurological disease conditions. 

### 4.1. VD and Alzheimer’s Disease (AD)

AD is one of the most common, devastating neurodegenerative disorders, with progressive deficit of memory and cognition dysfunction affecting elderly people around the world. Accumulated extracellular senile plaques and intracellular neurofibrillary tangles in the brain are the main hallmarks of AD. Senile plaques, made up of the aggregation of amyloid beta, are considered an early event in the pathogenesis of AD. At the molecular level, oxidative stress, mitochondrial dysfunction, inflammation, impaired neurotransmission, and metabolic changes are measured as a significant cause of AD onset and its pathogenesis. From this point of view, a medication that reduces these factors may have a potency in blocking the onset and progression of this disease. Currently, donepezil, the only drug available on the market, is used for the cure of AD pathogenesis, but it has some limitations because of the action on AchE level normalization only. AD is a multifactorial disease that suggests that optimization of a single factor is not sufficient for efficient therapy. This advocates the reason for the failure of donepezil and raises the concern of searching for new effective drugs for the complete cure of AD pathogenesis. On consideration of these points, studies suggested that drugs that have efficacy in acting on more than one factor might be more efficient as therapy. In this regard, the antioxidative, anti-inflammatory, and anti-apoptotic nature of VD has been confirmed with different doses at the preclinical level in multiple studies. A study confirmed that 100 µg/kg VD supplementation for 4 weeks in mice significantly reduces Aβ aggregation by increasing Nrf2 and HO-1 expression [32]. Another study by Karin et al., 2020 [54], suggested that VD deficiency positively correlated with the induction of oxidative stress, inflammation, and apoptosis. VD administration twice a day for 4 weeks at a 1 µg/kg dose reduced MDA content and IL-6, Aβ, and tau protein levels in the LPS-induced rat model of AD [55]. Improved mitochondrial complexes activity, decreased NFkB, IL-1β, IL-6, and TNF-α levels, and reduced microglial activation were reported after 42 IU/kg VD administration in STZ-induced AD in rats [56]. Studies confirmed that oxidative stress and inflammation are the primary biochemical changes that have been linked with survival, synaptic transmission, and memory deficit in AD. In this line, VD also acts on the reduction of survival, synaptic transmission, and memory deficit in AD. A study has confirmed that 1000 and 10,000 IU/kg VD administration for 2 weeks significantly normalizes the alteration in memory related to Morris water maze behavior by the enhancement of BDNF expression in the hippocampus of STZ-induced AD rat brains [57]. Another also suggested that 500 IU/kg VD supplementation in the AD rats increases BDNF, SOD, CAT, and caspase-3 protein expressions [58]. The level of acetylcholine neurotransmitter, vital for memory formation, is reduced in the brain of AD because of over-activation of the acetylcholine esterase enzyme [59]. Administration of 42 and 125 µg/kg of VD in an in vivo model of AD rats significantly restored the level of acetylcholine by downregulating the overactive action of acetylcholine esterase enzyme [60]. Furthermore, in the in vitro cell line studies represented, VD reduced the Aβ-induced cell death mechanism in various cellular models of AD, such as mouse N2A cells, SH-SY5Y cells, and PC12 cells. Studies in mice cortical primary neuronal cell models suggested that VD at 1 nm dose for 48 and 72 h enhanced the activity of SOD, CAT, aGST, GSH, and NGF levels [61]. VD exposure to N2A cells at 100 nm reduced Aβ and processing enzymes [62]. It blocks the enhanced expression of the intrinsic mitochondrial component of apoptosis-related proteins [40]. VD decreases the release of cyt c protein from mitochondria to cytosol and promotes cell survival. Decreased Bax, caspase-3, and parp-1 protein expressions were examined in the in vitro study after VD supplementation. Overall, the above facts reveal that VD offers an efficient action to halt AD pathogenesis by its pleiotropic effect.

### 4.2. VD and Parkinson’s Disease (PD)

Parkinson’s disease is the most common neurogenerative disease next to AD, affecting 1–2% of people above the age of 65 years. It is mainly characterized by motor and non-motor neuron symptoms, including rigidity, bradykinesia, amnesia, depression, etc. Clinically, it is diagnosed by the reduction in dopamine levels because of the loss of dopaminergic neurons in the substantia nigra region of the brain [63]. Oxidative stress, mitochondrial function alteration, impaired dopamine metabolism, inflammation, and excitotoxicity are considered the possible etiology of PD [64]. Currently, a number of therapeutic drugs have been available for managing PD patients on the preclinical level, but at the clinical level, limited drugs have been studied. Levodopa, one of the most popular drugs, has been used for the treatment of PD and has some limitations. In spite of better efficacy, it is also noteworthy that it does not reduce the progression of the disease and symptomatic and non-symptomatic motor disability. Because of multiple side effects, some other drugs, such as MAO inhibitors, have been used for the reduction of Levodopa toxicity in PD patients. In this context, the search for effective drugs to cure the disease is still needed. In experimental PD models, VD has been represented to exert a neuroprotective effect in catalepsy animal models treated with rotenone and a 6-OHDA model [65]. VD mitigates the alteration in motor and cognitive function by inhibiting the phosphorylation and aggregation of SNCA proteins [66]. In a mouse model of PD, VD slows down cognitive alterations after exposure to the 6-OHDA model [67]. It normalizes the motor coordination function of PD, confirmed by the rotarod, open field experiments with enhancement of TH, DAT, and antioxidant proteins. Five to six months of VD supplementation at doses of 100, 1000, and 10,000 IU/kg ameliorated the haloperidol-induced impairment in learning and spatial type of memory assessed by the water maze in a rat model [68]. Supplementation of VD at 5–20 µM dose for 24 h has the potency to modulate the autophagy by the activation of the AMPK pathway and block mitochondrial function alteration and its related neurodegeneration mechanism in rotenone-exposed SH-SY5Y cells [69]. These results indicate that VD could be a potential therapeutic alternative for PD and illustrate the need for further research.

### 4.3. VD and Huntington’s Disease (HD)

HD is an autosomal-dominant inherited neurogenerative disorder caused by the CAG sequence expansion in exon 1 of the huntingtin gene. It is an early-onset disorder generally occurring in people 30–40 years of age. Clinically, it is diagnosed by the accumulation of aggregated htt proteins to itself or with other cellular proteins in the brain that eventually impair normal neuronal function, which eventually leads to the loss of neurons [70]. According to accumulating reports, it initially affects the striatum region of the brain. After that, the cortex region is found to be affected. Oxidative stress, inflammation, and mitochondrial function alteration are suggested as a possible cause of neuronal damage and loss of neurons in HD [71]. No effective drug has been available to cure the progression of HD pathogenesis. From this point of view, the search for an effective drug to halt HD symptoms is still needed. There are not many studies on understanding the effect of VD on HD at the preclinical level, but some available reports show VD as a notable neuroprotective compound in in vivo, in vitro, and alternative models of HD studies [72]. Striatal cells extracted from the 3-NP-infused mouse model of HD brain showed a significantly improved locomotory deficit by the normalization of SOD, CAT, and GPx antioxidant genes and survival BDNF and NGF gene expression after 500 IU VD supplementation for 15 days. Another study also suggested that a higher dose of VD administration (12,000 IU/kg for 5 days per week) significantly increases the survival of N171-82Q HD transgenic mice, but no effect was observed on motor-related impairments [13]. The conclusive result of these studies suggested that VD might have shown effective action to slow down HD pathogenesis, but more studies are still needed to better illustrate its positive actions.

### 4.4. VD and Amyotrophic Lateral Sclerosis (ALS)

ALS is a fatal neurological disorder with an elusive etiology, characterized by upper and lower motor neuron damage. According to the previous literature, oxidative stress, neurochemical imbalance, mitochondrial defects, and genetic mutations seem to be possible causes of ALS [73]. Currently, no drugs are available on the market to cure this disease. In this context, research is going on in the same field, and drugs are tested to pave the effective remedy. However, studies confirm VD level reduction in ALS, but studies on VD supplementation-mediated regulation of ALS pathology are very limited [74]. In a genetic mouse model of ALS, the model confirms that VD supplementation at 1 IU/g for 113 days reduces the early severity of disease and increases the time of disease onset [75]. In addition, supplementation of a low VD diet increases the vulnerability and progression of disease in the G93A mouse. In culture neurons, the active form of VD addition at a 100 nm dose significantly potentiated the neurotrophic factor and reduced the Fas-induced death in cells [76]. Another study suggests VD supplementation at 10 and 50 IU/g/day reduces motor deficit and muscle weakness in the genetic model of mice [77]. In this regard, the above studies conclude that VD might represent a positive effect on ALS pathogenesis in different models and need the attention of researchers to conduct more studies to better understand VD use as a better therapeutic option for ALS in the future.

### 4.5. VD and Schizophrenia

Schizophrenia is a neuropsychiatric disorder, essentially characterized by hallucination, delusion, anxiety, forgetfulness, and social withdrawal. Clinically, it is only diagnosed by an imbalance in neurotransmitter levels in the different regions of the brain. A number of studies have also classified this disease at later stages in the neurodevelopment disorder category. The factors associated with a rise in symptoms of SCZ are oxidative stress, inflammation, mitochondrial dysfunction, and neurotrophin level impairment. A number of antipsychotic drugs are used for the reduction of SCZ symptoms, but because of some limitations, research is still needed for an effective drug search. In this regard, VD grabs attention as an effective intervention against SCZ because of its significant potency in the regulation of risk factor modulation and normal brain health. Studies confirm VD deficiency in SCZ at both preclinical and clinical levels. A study by Nwosu and Kum-Nji, 2018, suggested VD level imbalance in the serum of children 3–17 years of age by secondhand exposure to nicotine administration [78]. Studies also confirmed the association of glutaminergic and dopamine receptor gene modulation with VD deficiency in a number of schizophrenic models [79]. A study by Wu et al., 2021, confirmed that 10,000 IU/kg VD administration in C57BL/6 mice significantly reduced anxiety levels by modulating the NR2A subunit of the glutaminergic receptor after nicotine exposure [8]. To reach a directional conclusion in understanding the effect of VD administration in the context of schizophrenia disease reduction at the preclinical level, more studies are still needed.

**Table 1:** Different doses of vitamin D used in preclinical studies of neurodegenerative diseases.

Disease Type	Experimental Model	Administered Dose	Mechanism	References
Alzheimer’s disease	Albino rat	1 µg/kg, 4 weeks	Nrf2, HO protein GSH, TNFα, GFAP, Ib-1, IL-10, MAPK, Erk, Tau, Aβ protein expression	[80]
	Wistar rat	42 IU/kg, 7 days	SOD, CAT activity, GSH level TNFα, NFkB protein, MDA, nitrite content level, AchE activity	[56]
	Wistar rat	1 µg/kg, 14 days	SOD activity, working memory MDA content, cresyl violet H&E-positive cells	[81]
	Wistar rat	100, 1000, 10,000 IU/kg, 3 weeks	BDNF level, SOD, CAT activity MDA, NO, nitrite content	[57]
	Wistar rat	5 μg/kg/day, 2 weeks	TAC, thiol, spatial memory, DNA damage, MDA content	[12]
	SH-SY5Y cells and ICR mice	10% CCE in drinking water, 21 days	VDR, MTHFR, LCMT-1, PP2A, p-TAU (Thr396), T-TAU protein expression, methylated PP2A gene expression	[82]
	APPswe/PS1dE9	10,000, 12,000 IU/kg	Aβ, GFAP	[83]
	5XFAD mice model	500, 1000, 7500 IU/kg, weeks	Aβ, GFAP, MAP2 and nestin protein levels	[84]
	5XFAD mice model	1000, 7500 IU/kg, 4 weeks	Aβ, Iba1 protein Level	[33]
	Albino mice	100 µg/kg, 4 weeks	Nrf2, HO, SYP, SIRT-1, PD-95 protein levels NF-kB, TNF-α, IL-1β protein levels	[32]
	Kung Ming mice	2.5 µg/kg, 14 days	Aβ, LRP, RAGE, and VDR protein levels	[85]
	Wistar rat	1000 IU/kg	Nrf2 and SOD, CAT activity, GSH level NF-kB, TNF-α, IL-10 protein expression, MDA content, and ROS level	[86]
	APPswe/PS1dE9 mice	10,000 IU/kg	Aβ, Iba1 protein Level	[87]
	OVX rats	1.0, 2.5, or 5.0 mg/kg, 14 days	Aβ, CAT activity, GSH level	[88]
	Primary cortical neuronal cell culture	1 nM, 72 h	SOD, CAT, GST activity and GSH level, NGF protein expression	[61]
	Longe Evans hooded rats and CD1 mice, SHSY5Y	100 nM, 24 h	Sphingosine kinase activity Aβ, S1P)/ceramide, p38MAPK/ATF4 protein expression	[89]
Parkinson’s disease	SH-SY5Y	01, 0.5, 1, 5, or 10 nM	Calbindin-D28k (CB) and a-syn	[90]
	SH-SY5Y	2.5 lM, 5 lM, and 10 lM for 2 h	LC3, beclin-1, AMPK, caspase-3, bak, Bax, beclin, and mTOR protein expression	[69]
	C57BL/6 mice	0.2, 1, and 5 μg/kg for 7 days	TH, LCII, beclin mTOR, P62 level	[91]
	HN9.10e cells	100 nM	Cadherin, VDR protein level GFAP, PPARγ	[40]
	Mice	1 µg/kg/day, 10 days	TH GFAP, Iba-1, TLR4, iNOS, TGF-β, IL-4, IL10, CD206, CD163, CD204 protein levels	[92]
	Mice	1 μg/kg/day, 10 days	TH GFAP, Iba-1, TLR4, iNOS, TGF-β, IL-4, IL- 10, CD206, CD163, CD204 protein levels	[61]
	Mice	30 mg/kg, 7 days	TH, DAT, BDNF, MAO-B CD11b, IL-I β, and p47phox protein levels	[67]
	Wistar rat	1 μg/kg VD3 for 7 days	TH, DAT, VDR3, DA, DOPAC levels, TNF-α expression and nitrite, TBARS content Level	[93]
	Wistar rat	100, 1000, 10,000 IU/kg, 6 months	Synaptogenein 1, 2 expression	[68]
	Wistar rat	1.0 μg/kg, 8 days	DA, DOPAC, HVA levels	[65]
	Wistar rat	1 μg/kg/day, 14 days	Respiratory consumption, SOD activity, TH, DAT, VDAC and HSP60 protein Levels H_2_O_2_ production	[43]
Huntington’s disease	N171-82Q	12,000 IU/kg, 101 days	Lifespan	[13]
Amyotrophic lateral sclerosis	G93A mouse	1 IU/g, 113 days	No change in grip strength, movement motor activity	[75]
	Primary cortical neuron	100 nM	BDNF expression Fas, caspase-3 protein expression	[76]
Schizophrenia	C57BL/6 mice	10,000 IU/kg, 42 days	α7 nAChR NR2A	[8]

Abbreviations: Nrf2 = nuclear factor erythroid 2-related factor 2, HO = heam-oxygenase, α7 nAChR = alpha7 acetylcholine receptor, BDNF = brain-derived growth factor, Hsp60 = heat shock protein 60, DAT= dopamine transporter, VDAC = voltage-dependent anion channels, SOD = sodium oxidase dismutase, TH = thyroxine hydroxylase, DA = dopamine, DPAC = 3,4-dihydroxyphenylacetic acid, GSH = reduced glutathione, TNFα = tumor necrosis factor, GFAP = glial fibrillary acidic protein, Iba-1 = ionized calcium-binding adapter molecule 1, IL-10 = interleukin 10, MAPK = map-associated protein kinase, Erk = extracellular signal-regulated kinases, Aβ = amyloid beta, NR2A = NMDA receptor 2A, H_2_O_2_ = hydrogen peroxide, GDNF = growth-derived nerve growth factor, MAO1 = monoamine oxidase1, CD11b = cell death receptor 11b, TLR4 = toll-like receptor4, NFkB= nuclear factor kappa-light-chain-enhancer of activated B cells, MDA= malondialdehyde, AchE = acetylcholine receptor esterase, PPAR = peroxisome proliferator-activated receptor, VDR = vitamin D receptor, PD-95 = postsynaptic density protein-95, SIRT1 = silent mating-type information regulation 2 homolog 1, mTOR = mammalian target of rapamycin, LC3 = microtubule-associated protein 1A/1B-light chain 3, AMPK = AMP-activated protein kinase, RAGE = receptor for advanced glycation end products.

## 5. Clinical Relevance of VD’s Role in Neurodegenerative Diseases

Clinical evidence assessing the effect of VD supplementation ushered new hope in paving effective drugs that have the potency to reduce the symptoms arising in neurodegenerative disorders. A number of studies have shown the positive effect of VD and have drawn researchers’ attention to exploring more (Table 2). Some clinical studies also confirm that VD has no influence on the course of neurological diseases with an absence of any side effects. In this section, we summarize information on the available clinical evidence of VD implemented in different neurological disorders to better comprehend VD use as a remedy.

### 5.1. AD

Evidence at the clinical level to check the efficacy of VD effect in AD patients is very limited. Besides the limited literature on the VD effect in AD patients, a number of studies have confirmed that AD patients suffer from lower VD levels [94]. A study of randomized control, double = blind, and placebo-controlled trials confirmed that oral administration of VD in combination with memantine for 24 weeks improved cognitive performance in AD patients [95]. A recent study suggested that 6-month VD supplementation normalizes cognitive impairment and reduces oxidative damage in 16 MCI patients.

### 5.2. PD

A study by Suzuki et al., 2013, suggested that VD supplementation at a 1200 IU dose per day for a period of 12 months showed enhancement in the motor scores (H&Y stage, UPDRS) for a short period in 58 patients [96]. A prominent phenotype visualized in PD patients is balance problems and falls, which are major challenges to cure, even in the presence of many non-pharmacological therapies against this type of deficit. A recent study suggested that VD at a 10,000 IU/day administration for 16 weeks showed a positive effect on balance deficit in a randomized, double-blind intervention trial of 58 patients.

### 5.3. HD

In the context of other neurodegenerative diseases, there are no studies available on VD supplementation in HD patients. Besides the absence of VD supplementation studies, VD insufficiency has been confirmed in HD patients. Evidence of a positive correlation of VD insufficiency with cognitive and memory-related symptoms might suggest the use of VD, due to better efficacy, as a potential candidate to reduce the course of pathogenesis in HD patients.

### 5.4. ALS

Cumulative results of research studies have confirmed that ALS patients who suffer from VD deficiency and VD supplementation are involved in the reduction of cognitive decline. According to Karam et al., 2013, they reported that approximately 81% of patients with ALS suffered from reduced VD levels, and after 2000 IU supplementation of VD for 9 months, they showed a significant change in the Amyotrophic Lateral Sclerosis Functional Rating Scale (ALSFRS-R) score [97]. Another study suggested that VD deficiency has been associated with the progression of disease and survival in an ALS patient cohort [76]. Cohort data of 33 ALS patients showed an improvement in VD serum levels after 75 and 100 IU VD supplementation for the month [98]. Some studies also describe VD as having no effect on ALS patients. In a study by Libonati et al., 2017, it was suggested that VD did not show any changes in ALSFRS score in 42–87-year-old male and female ALS patients [97]. Cumulative results of the available studies advocate that VD might have the potency to alleviate symptoms of ALS and strengthen the need for more clinical studies to better understand the VD effect.

### 5.5. Schizophrenia

In addition to the preclinical level, researchers also confirm the association of VD deficiency with neuropsychiatric disorders, and this grabs their attention to validate the VD effect in neuropsychiatric patients. A mixed interpretation of meta-analysis, clinical trials, and systemic studies are discussed here. According to Ghaderi et al., 2019, after 12-week VD interventions (50,000 IU) in schizophrenic patients (*n* = 60), the VD level in plasma increased, normalizing the impaired PANSS score. VD mediated a reduction in MDA and increased the serum high-sensitivity C-reactive protein (hs-CRP) levels reported in the plasma of patients [99]. This study also confirmed the metabolic profiling alteration in the same patient, which was also significantly normalized by VD supplementation.

According to Fond et al., 2018, VD supplementation for 12 months also showed potency to reduce anxiety- and depression-like changes in schizophrenic patients [100]. On the other hand, according to Thakurathi et al., 2013, a lower dose of VD administration (400 IU) for 7 months did not show any changes in symptoms of schizophrenic patients [101]. In addition to lower doses, some studies have suggested that short-term exposure might not have shown an effect in schizophrenic patients [14]. In this regard, the data produced from the available studies at the clinical level advocate the beneficial effect of VD to some extent, but before reaching any final conclusion, there is a need for more studies to explore the VD effect in schizophrenic in the direction of an effective drug search, which can be used in the future as a remedy.

**Table 2:** Clinical studies demonstrating neuroprotective effects of vitamin D.

Disease Type	Study Criteria	Outcome	Reference
Alzheimer’s disease	40 healthy Canadian adults aged 50–60 (4000 and 400 IU/day, 18 weeks follow-up)	Improvement in visual memory but no change in verbal memory at higher doses	[94]
	16 MCI, 11 VEAD, 25 healthy patients evaluated (18-month follow-up)	After 6-month VD supplementation, Aβ1-40 level was normalized, and long-term treatment reduced the risk of cognitive decline	[102]
	130 healthy African American postmenopausal women, trial intervention Vit D3 (2400 IU–3600 IU or 4800 IU/day, follow-up 3 years)	No difference in cognition over time in older African American women	[103]
	120 patients, aged 60 years and older, participated (100,000 IU, every 4 weeks for 24 weeks)	Improvement in mental status and cognitive state, normalized Ca^+2^ and PTH levels	[80]
	210 AD patients, 65 years and older, Chinese population (800 IU/kg for 6 and 12 months)	Aβ42, APP, BACE1 mRNA levels in the plasma	[95]
Parkinson’s disease	51 randomized participants aged 52–66 and 67–86 years (10,000 IU/day, 16 weeks)	Higher dose is safe for the short term, with no significant change observed in the balance in older patients but improvement in the younger patients	[104]
	114 PD patients aged 45–85 years (1200 IU/day 12 months)	Significantly prevented PD deterioration, analyzed by the HY stage and UODRS score without inducing hypocalcemia	[80]
	209 northern China patients aged 64.6 ± 9.4 years (different doses of VD administration 0–5 μg/d, 5–8 μg/d, 8–12 μg/d, >12 μg/d)	Outdoor activity and VD intake reduce the risk of PD	[105]
Huntington’s Disease	No evidence found	No evidence found	No evidence found
ALS	48 ALS patients, 34 with deficient (<20 ng/mL) and 14 with insufficient (20–29 ng/mL) serum levels of 25(OH)D (50.000, 75.000, and 100.000 international units (IU)/month for 6 months)	No effect observed on motor alteration, but significant increase in VD serum level was detected in 75.000 and 100.000 IU doses	[83]
	688 participants, aged 70 years and older (200, 1000, 2000, or 4000 IU of vitamin D_3_ per day)	No difference observed on fall or death	[106]
	57 normal and 57 patients, aged 42–82 years old (100,000 every 3-month follow-up)	No better prognosis observed in relation to untreated ALS patients	[85]
Schizophrenia	18–65 years old, Clozapine-treated participants (14,000 IU VD supplemented for two weeks)	Improved cognition but no effect on psychosis, mood, and metabolic profiling	[107]
	Total 149 (18–65-year-olds) were randomized, and 104 were followed up to 6 months after the screening (12,000 IU/month VD-supplemented)	No difference observed in mental health and metabolic profiling	[108]
	18 and 65 years old, including women of child-bearing age, were included (120,000 IU/month for 6 months)	Improved early psychosis-related mental alteration	[109]
	60 patients with chronic schizophrenia participated (50,000 IU vitamin D3 supplementation every 2 weeks in combination with probiotic for 12 weeks)	Beneficial effects observed on the general and total PANSS scores and metabolic profiles	[86]

Abbreviation: PTH = parathyroid hormone, Aβ42 = amyloid beta-42, APP = amyloid precursor protein, BACE1 = beta-secretase 1, UODRS = unified Parkinson’s disease rating scale, PANSS = positive and negative syndrome scale.

## 6. Conclusions

The review discusses the potential role of vitamin D as a promising neuroprotective agent in addressing brain-related disorders. It highlights how low vitamin D levels are associated with several detrimental conditions, such as oxidative stress, inflammation, apoptosis (cell death), and reduced levels of neurotrophins, which are essential for neuron survival and function. These factors are commonly implicated in various neurological diseases, including Alzheimer’s, Parkinson’s, and Huntington’s disease. This review emphasizes that vitamin D can help reduce oxidative stress and inflammation, both of which are major contributors to neurodegeneration. Additionally, it promotes cell survival by decreasing apoptosis and enhancing neurotrophin levels, which support neuronal growth and cognitive function. By compiling evidence from in vitro, in vivo, and clinical studies, the review suggests that vitamin D has the potential to mitigate neurodegenerative diseases and improve overall brain health, but the VD effect on brain-related disorders of newborn babies and pregnant women is still concerning. More studies are still needed to bring clarity to future results. Thus, vitamin D emerges as a valuable intervention with significant implications for preventing or treating neurological conditions.

## 7. Future of VD as a Neuroprotective Agent

The above evidence suggests that VD could be useful for counteracting age-related processes, and clinical reports have entailed that VD has positive effects on cognition. A better comprehension of how VD behaves may help researchers who work in neuroscience plan future experiment-related trials with success. VD behaves like a neuroprotective agent by involving multi steps of energy production with less amount of ROS generation and inflammatory molecule release. It plays a significant role in the reduction of oxidative stress, microglial activation, and apoptosis-related protein activation. Accumulating evidence suggests that VD is involved in the slowing of many signaling processes in the brain related to oxidative stress, inflammation, apoptosis, and neurogenesis. The area of aging-related research in the neuroscience field is required for the emergence of a cure and prevention of these disorders. In this context, the much higher value of VD at the current time might up its potency in treating and decoding underlying mechanisms responsible for neurodegeneration.

## References

[1] Cutolo M., Paolino S., Sulli A., Smith V., Pizzorni C., Seriolo B. (2014). Vitamin D, steroid hormones, and autoimmunity. Ann. N. Y. Acad. Sci..

[2] Eyles D.W. (2021). Vitamin D: Brain and Behavior. JBMR Plus.

[3] Farghali M., Ruga S., Morsanuto V., Uberti F. (2020). Can Brain Health Be Supported by Vitamin D-Based Supplements? A Critical Review. Brain Sci..

[4] Bivona G., Gambino C.M., Iacolino G., Ciaccio M. (2019). Vitamin D and the nervous system. Neurol. Res..

[5] Sultan S., Taimuri U., Basnan S.A., Ai-Orabi W.K., Awadallah A., Almowald F., Hazazi A. (2020). Low Vitamin D and Its Association with Cognitive Impairment and Dementia. J. Aging Res..

[6] Utkan Karasu A., Kaymak Karataş G. (2021). Effect of vitamin D supplementation on lower extremity motor function and ambulation in stroke patients. Turk. J. Med. Sci..

[7] Moretti R., Morelli M.E., Caruso P. (2018). Vitamin D in Neurological Diseases: A Rationale for a Pathogenic Impact. Int. J. Mol. Sci..

[8] Wu B., Tao X., Liu C., Li H., Jiang T., Chen Z., Wang Q., Liu F., Mu M., Chen Z. (2021). Vitamin D3 reduces hippocampal NR2A and anxiety in nicotine withdrawal mice. Transl. Neurosci..

[9] Karampela I., Sakelliou A., Vallianou N., Christodoulatos G.-S., Magkos F., Dalamaga M. (2021). Vitamin D and Obesity: Current Evidence and Controversies. Curr. Obes. Rep..

[10] Zhu C., Zhang Y., Wang T., Lin Y., Yu J., Xia Q., Zhu P., Zhu D.M. (2020). Vitamin D supplementation improves anxiety but not depression symptoms in patients with vitamin D deficiency. Brain Behav..

[11] Jiang J., Shi D., Zhou X.-Q., Yin L., Feng L., Jiang W.-D., Liu Y., Tang L., Wu P., Zhao Y. (2015). Vitamin D inhibits lipopolysaccharide-induced inflammatory response potentially through the Toll-like receptor 4 signalling pathway in the intestine and enterocytes of juvenile Jian carp (*Cyprinus carpio* var. Jian). Br. J. Nutr..

[12] Mehri N., Haddadi R., Ganji M., Shahidi S., Soleimani Asl S., Taheri Azandariani M., Ranjbar A. (2020). Effects of vitamin D in an animal model of Alzheimer’s disease: Behavioral assessment with biochemical investigation of Hippocampus and serum. Metab. Brain Dis..

[13] Molnar M.F., Török R., Szalárdy L., Sümegi E., Vécsei L., Klivényi P. (2016). High-dose 1,25-dihydroxyvitamin D supplementation elongates the lifespan of Huntington’s disease transgenic mice. Acta Neurobiol. Exp..

[14] Tiangga E., Gowda A., Dent J.A. (2008). Vitamin D deficiency in psychiatric in-patients and treatment with daily supplements of calcium and ergocalciferol. Psychiatr. Bull..

[15] Bikle D.D. (2014). Vitamin D metabolism, mechanism of action, and clinical applications. Chem. Biol..

[16] Christakos S., Dhawan P., Verstuyf A., Verlinden L., Carmeliet G. (2016). Vitamin D: Metabolism, Molecular Mechanism of Action, and Pleiotropic Effects. Physiol. Rev..

[17] Jones G., Prosser D.E., Kaufmann M. (2014). Cytochrome P450-mediated metabolism of vitamin D. J. Lipid Res..

[18] Smolders J., Schuurman K.G., van Strien M.E., Melief J., Hendrickx D., Hol E.M., van Eden C., Luchetti S., Huitinga I. (2013). Expression of vitamin D receptor and metabolizing enzymes in multiple sclerosis-affected brain tissue. J. Neuropathol. Exp. Neurol..

[19] Gáll Z., Székely O. (2021). Role of Vitamin D in Cognitive Dysfunction: New Molecular Concepts and Discrepancies between Animal and Human Findings. Nutrients.

[20] Di Somma C., Scarano E., Barrea L., Zhukouskaya V.V., Savastano S., Mele C., Scacchi M., Aimaretti G., Colao A., Marzullo P. (2017). Vitamin D and Neurological Diseases: An Endocrine View. Int. J. Mol. Sci..

[21] Rotermund C., Machetanz G., Fitzgerald J.C. (2018). The Therapeutic Potential of Metformin in Neurodegenerative Diseases. Front. Endocrinol..

[22] Franco-Iborra S., Vila M., Perier C. (2016). The Parkinson Disease Mitochondrial Hypothesis: Where Are We at?. Neurosci. Rev. J. Bringing Neurobiol. Neurol. Psychiatry.

[23] Andersen J.K. (2004). Oxidative stress in neurodegeneration: Cause or consequence?. Nat. Med..

[24] Ibi M., Sawada H., Nakanishi M., Kume T., Katsuki H., Kaneko S., Shimohama S., Akaike A. (2001). Protective effects of 1 alpha,25-(OH)_2_D_3_ against the neurotoxicity of glutamate and reactive oxygen species in mesencephalic culture. Neuropharmacology.

[25] Tang H., Hua F., Wang J., Yousuf S., Atif F., Sayeed I., Stein D.G. (2015). Progesterone and vitamin D combination therapy modulates inflammatory response after traumatic brain injury. Brain Inj..

[26] Mello-Filho A.C., Meneghini R. (1991). Iron is the intracellular metal involved in the production of DNA damage by oxygen radicals. Mutat. Res..

[27] Chen K.-B., Lin A.M.-Y., Chiu T.-H. (2003). Systemic vitamin D3 attenuated oxidative injuries in the locus coeruleus of rat brain. Ann. N. Y. Acad. Sci..

[28] Lin M.T., Beal M.F. (2006). Mitochondrial dysfunction and oxidative stress in neurodegenerative diseases. Nature.

[29] Hajiluian G., Abbasalizad Farhangi M., Nameni G., Shahabi P., Megari-Abbasi M. (2018). Oxidative stress-induced cognitive impairment in obesity can be reversed by vitamin D administration in rats. Nutr. Neurosci..

[30] Cui C., Wang C., Jin F., Yang M., Kong L., Han W., Jiang P. (2021). Calcitriol confers neuroprotective effects in traumatic brain injury by activating Nrf2 signaling through an autophagy-mediated mechanism. Mol. Med. Camb. Mass.

[31] Domenico P., Guido P., Carlo M., Sara L., Serenella S., De Stefano N. (2023). Vitamin D in Neurological Diseases. Int. J. Mol. Sci..

[32] Ali Ammar Shah S.A., Zaman N., Uddin M.N., Khan W., Ali Abid Riaz M., Kamil A. (2021). Vitamin D exerts neuroprotection via SIRT1/nrf-2/NF-kB signaling pathways against D-galactose-induced memory impairment in adult mice. Neurochem. Int..

[33] Landel V., Millet P., Baranger K., Loriod B., Féron F. (2016). Vitamin D interacts with Esr1 and Igf1 to regulate molecular pathways relevant to Alzheimer’s disease. Mol. Neurodegener..

[34] Rao Z., Chen X., Wu J., Xiao M., Zhang J., Wang B., Fang L., Zhang H., Wang X., Yang S. (2019). Vitamin D Receptor Inhibits NLRP3 Activation by Impeding Its BRCC3-Mediated Deubiquitination. Front. Immunol..

[35] Radi E., Formichi P., Battisti C., Federico A. (2014). Apoptosis and oxidative stress in neurodegenerative diseases. J. Alzheimer’s Dis. JAD.

[36] Lin A.M.Y., Chen K.B., Chao P.L. (2005). Antioxidative effect of vitamin D3 on zinc-induced oxidative stress in CNS. Ann. N. Y. Acad. Sci..

[37] Bao Z., Wang X., Li Y., Feng F. (2020). Vitamin D Alleviates Cognitive Dysfunction by Activating the VDR/ERK1/2 Signaling Pathway in an Alzheimer’s Disease Mouse Model. Neuroimmunomodulation.

[38] Lee T.-L., Lee M.-H., Chen Y.-C., Lee Y.-C., Lai T.-C., Lin H.Y.-H., Hsu L.-F., Sung H.-C., Lee C.-W., Chen Y.-L. (2020). Vitamin D Attenuates Ischemia/Reperfusion-Induced Cardiac Injury by Reducing Mitochondrial Fission and Mitophagy. Front. Pharmacol..

[39] Wang J.-W., Zhu L., Shi P.-Z., Wang P.-C., Dai Y., Wang Y.-X., Lu X.-H., Cheng X.-F., Feng X.-M., Zhang L. (2020). 1,25(OH)_2_D_3_ Mitigates Oxidative Stress-Induced Damage to Nucleus Pulposus-Derived Mesenchymal Stem Cells through PI3K/Akt Pathway. Oxid. Med. Cell. Longev..

[40] Cataldi S., Arcuri C., Hunot S., Mecca C., Codini M., Laurenti M.E., Ferri I., Loreti E., Garcia-Gil M., Traina G. (2018). Effect of Vitamin D in HN9.10e Embryonic Hippocampal Cells and in Hippocampus from MPTP-Induced Parkinson’s Disease Mouse Model. Front. Cell. Neurosci..

[41] Ricca C., Aillon A., Bergandi L., Alotto D., Castagnoli C., Silvagno F. (2018). Vitamin D Receptor Is Necessary for Mitochondrial Function and Cell Health. Int. J. Mol. Sci..

[42] Britti E., Delaspre F., Sanz-Alcázar A., Medina-Carbonero M., Llovera M., Purroy R., Mincheva-Tasheva S., Tamarit J., Ros J. (2021). Calcitriol increases frataxin levels and restores mitochondrial function in cell models of Friedreich Ataxia. Biochem. J..

[43] Araújo de Lima L., Oliveira Cunha P.L., Felicio Calou I.B., Tavares Neves K.R., Facundo H.T., Socorro de Barros Viana G. (2020). Effects of vitamin D (VD3) supplementation on the brain mitochondrial function of male rats, in the 6-OHDA-induced model of Parkinson’s disease. Neurochem. Int..

[44] Yuan J., Guo X., Liu Z., Zhao X., Feng Y., Song S., Cui C., Jiang P. (2018). Vitamin D receptor activation influences the ERK pathway and protects against neurological deficits and neuronal death. Int. J. Mol. Med..

[45] Huang Y.-N., Ho Y.-J., Lai C.-C., Chiu C.-T., Wang J.-Y. (2015). 1,25-Dihydroxyvitamin D3 attenuates endotoxin-induced production of inflammatory mediators by inhibiting MAPK activation in primary cortical neuron-glia cultures. J. Neuroinflamm..

[46] Wimalawansa S.J. (2019). Vitamin D Deficiency: Effects on Oxidative Stress, Epigenetics, Gene Regulation, and Aging. Biology.

[47] Matthes F., Hettich M.M., Schilling J., Flores-Dominguez D., Blank N., Wiglenda T., Buntru A., Wolf H., Weber S., Vorberg I. (2018). Inhibition of the MID1 protein complex: A novel approach targeting APP protein synthesis. Cell Death Discov..

[48] Cui C., Cui J., Jin F., Cui Y., Li R., Jiang X., Tian Y., Wang K., Jiang P., Gao J. (2017). Induction of the Vitamin D Receptor Attenuates Autophagy Dysfunction-Mediated Cell Death Following Traumatic Brain Injury. Cell. Physiol. Biochem. Int. J. Exp. Cell. Physiol. Biochem. Pharmacol..

[49] Gezen-Ak D., Dursun E., Yilmazer S. (2011). The effects of vitamin D receptor silencing on the expression of LVSCC-A1C and LVSCC-A1D and the release of NGF in cortical neurons. PLoS ONE.

[50] Peitl V., Silić A., Orlović I., Vidrih B., Crnković D., Karlović D. (2020). Vitamin D and Neurotrophin Levels and Their Impact on the Symptoms of Schizophrenia. Neuropsychobiology.

[51] Orme R.P., Bhangal M.S., Fricker R.A. (2013). Calcitriol imparts neuroprotection in vitro to midbrain dopaminergic neurons by upregulating GDNF expression. PLoS ONE.

[52] Shirazi H.A., Rasouli J., Ciric B., Rostami A., Zhang G.-X. (2015). 1,25-Dihydroxyvitamin D3 enhances neural stem cell proliferation and oligodendrocyte differentiation. Exp. Mol. Pathol..

[53] Domanskyi A., Parlato R. (2022). Oxidative Stress in Neurodegenerative Diseases. Antioxidants.

[54] Karin A., Mario S., Magdalena H., Stefan N., Markus K., Adelina T.B., Gennaro M., Stefan P., Oliver M. (2020). Vitamin D deficiency 2.0: An update on the current status worldwide. Eur. J. Clin. Nutr..

[55] Medhat E., Rashed L., Abdelgwad M., Aboulhoda B.E., Khalifa M.M., El-Din S.S. (2020). Exercise enhances the effectiveness of vitamin D therapy in rats with Alzheimer’s disease: Emphasis on oxidative stress and inflammation. Metab. Brain Dis..

[56] Yamini P., Ray R.S., Chopra K. (2018). Vitamin D3 attenuates cognitive deficits and neuroinflammatory responses in ICV-STZ induced sporadic Alzheimer’s disease. Inflammopharmacology.

[57] Mansouri F., Ghanbari H., Marefati N., Arab Z., Salmani H., Beheshti F., Hosseini M. (2021). Protective effects of vitamin D on learning and memory deficit induced by scopolamine in male rats: The roles of brain-derived neurotrophic factor and oxidative stress. Naunyn. Schmiedebergs Arch. Pharmacol..

[58] Khairy E.Y., Attia M.M. (2021). Protective effects of vitamin D on neurophysiologic alterations in brain aging: Role of brain-derived neurotrophic factor (BDNF). Nutr. Neurosci..

[59] Khatoon R., Rasheed M.Z., Rawat M., Alam M.M., Tabassum H., Parvez S. (2019). Effect of melatonin on Aβ42 induced changes in the mitochondrial function related to Alzheimer’s disease in *Drosophila melanogaster*. Neurosci. Lett..

[60] Rodrigues M.V., Gutierres J.M., Carvalho F., Lopes T.F., Antunes V., da Costa P., Pereira M.E., Schetinger M.R.C., Morsch V.M., de Andrade C.M. (2019). Protection of cholinergic and antioxidant system contributes to the effect of Vitamin D3 ameliorating memory dysfunction in sporadic dementia of Alzheimer’s type. Redox Rep. Commun. Free Radic. Res..

[61] Alamro A.A., Alsulami E.A., Almutlaq M., Alghamedi A., Alokail M., Haq S.H. (2020). Therapeutic Potential of Vitamin D and Curcumin in an In Vitro Model of Alzheimer Disease. J. Cent. Nerv. Syst. Dis..

[62] Grimm M.O.W., Lehmann J., Mett J., Zimmer V.C., Grösgen S., Stahlmann C.P., Hundsdörfer B., Haupenthal V.J., Rothhaar T.L., Herr C. (2014). Impact of Vitamin D on amyloid precursor protein processing and amyloid-β peptide degradation in Alzheimer’s disease. Neurodegener. Dis..

[63] Claudio G., Silvia C., Fabio B. (2021). Potential therapeutic effects of polyphenols in Parkinson’s disease: In vivo and in vitro pre-clinical studies. Neural Regen Res..

[64] Khang R., Park C., Shin J.-H. (2015). Dysregulation of parkin in the substantia nigra of db/db and high-fat diet mice. Neuroscience.

[65] Cass W.A., Peters L.E., Fletcher A.M., Yurek D.M. (2014). Calcitriol promotes augmented dopamine release in the lesioned striatum of 6-hydroxydopamine treated rats. Neurochem. Res..

[66] Zhang Y., Ji W., Zhang S., Gao N., Xu T., Wang X., Zhang M. (2020). Vitamin D Inhibits the Early Aggregation of α-Synuclein and Modulates Exocytosis Revealed by Electrochemical Measurements. Angew. Chem. Int. Ed. Engl..

[67] Bayo-Olugbami A., Nafiu A.B., Amin A., Ogundele O.M., Lee C.C., Owoyele B.V. (2020). Vitamin D attenuated 6-OHDA-induced behavioural deficits, dopamine dysmetabolism, oxidative stress, and neuro-inflammation in mice. Nutr. Neurosci..

[68] Latimer C.S., Brewer L.D., Searcy J.L., Chen K.-C., Popović J., Kraner S.D., Thibault O., Blalock E.M., Landfield P.W., Porter N.M. (2014). Vitamin D prevents cognitive decline and enhances hippocampal synaptic function in aging rats. Proc. Natl. Acad. Sci. USA.

[69] Jang W., Park H.-H., Lee K.-Y., Lee Y.J., Kim H.-T., Koh S.-H. (2015). 1,25-dyhydroxyvitamin D3 attenuates L-DOPA-induced neurotoxicity in neural stem cells. Mol. Neurobiol..

[70] Sanchis A., García-Gimeno M.A., Cañada-Martínez A.J., Sequedo M.D., Millán J.M., Sanz P., Vázquez-Manrique R.P. (2019). Metformin treatment reduces motor and neuropsychiatric phenotypes in the zQ175 mouse model of Huntington disease. Exp. Mol. Med..

[71] Tabrizi S.J., Flower M.D., Ross C.A., Wild E.J. (2020). Huntington disease: New insights into molecular pathogenesis and therapeutic opportunities. Nat. Rev. Neurol..

[72] Rai S.N., Singh P., Steinbusch H.W.M., Vamanu E., Ashraf G., Singh M.P. (2021). The Role of Vitamins in Neurodegenerative Disease: An Update. Biomedicines.

[73] Faye P.A., Poumeaud F., Miressi F., Lia A.S., Demiot C., Magy L., Favreau F., Sturtz F.G. (2019). Focus on 1,25-Dihydroxyvitamin D3 in the Peripheral Nervous System. Front. Neurosci..

[74] Lương K.V.Q., Nguyễn L.T.H. (2013). Roles of vitamin D in amyotrophic lateral sclerosis: Possible genetic and cellular signaling mechanisms. Mol. Brain.

[75] Solomon J.A., Gianforcaro A., Hamadeh M.J. (2011). Vitamin D3 deficiency differentially affects functional and disease outcomes in the G93A mouse model of amyotrophic lateral sclerosis. PLoS ONE.

[76] Camu W., Tremblier B., Plassot C., Alphandery S., Salsac C., Pageot N., Juntas-Morales R., Scamps F., Daures J.-P., Raoul C. (2014). Vitamin D confers protection to motoneurons and is a prognostic factor of amyotrophic lateral sclerosis. Neurobiol. Aging.

[77] Gianforcaro A., Solomon J.A., Hamadeh M.J. (2013). Vitamin D(3) at 50x AI attenuates the decline in paw grip endurance, but not disease outcomes, in the G93A mouse model of ALS, and is toxic in females. PLoS ONE.

[78] Nwosu B.U., Kum-Nji P. (2018). Tobacco smoke exposure is an independent predictor of vitamin D deficiency in US children. PLoS ONE.

[79] Cui X., McGrath J.J., Burne T.H.J., Eyles D.W. (2021). Vitamin D and schizophrenia: 20 years on. Mol. Psychiatry.

[80] Saad El-Din S., Rashed L., Medhat E., Emad Aboulhoda B., Desoky Badawy A., Mohammed ShamsEldeen A., Abdelgwad M. (2020). Active form of vitamin D analogue mitigates neurodegenerative changes in Alzheimer’s disease in rats by targeting Keap1/Nrf2 and MAPK-38p/ERK signaling pathways. Steroids.

[81] Mehrabadi S., Sadr S.S. (2020). Administration of Vitamin D_3_ and E supplements reduces neuronal loss and oxidative stress in a model of rats with Alzheimer’s disease. Neurol. Res..

[82] Pan Y., Zhang Y., Liu N., Lu W., Yang J., Li Y., Liu Z., Wei Y., Lou Y., Kong J. (2021). Vitamin D Attenuates Alzheimer-like Pathology Induced by Okadaic Acid. ACS Chem. Neurosci..

[83] Morello M., Landel V., Lacassagne E., Baranger K., Annweiler C., Féron F., Millet P. (2018). Vitamin D Improves Neurogenesis and Cognition in a Mouse Model of Alzheimer’s Disease. Mol. Neurobiol..

[84] Wong D., Broberg D.N., Doad J., Umoh J.U., Bellyou M., Norley C.J.D., Holdsworth D.W., Montero-Odasso M., Beauchet O., Annweiler C. (2021). Effect of Memantine Treatment and Combination with Vitamin D Supplementation on Body Composition in the APP/PS1 Mouse Model of Alzheimer’s Disease Following Chronic Vitamin D Deficiency. J. Alzheimer’s Dis. JAD.

[85] Guo Y.X., He L.Y., Zhang M., Wang F., Liu F., Peng W.X. (2016). 1,25-Dihydroxyvitamin D3 regulates expression of LRP1 and RAGE in vitro and in vivo, enhancing Aβ1-40 brain-to-blood efflux and peripheral uptake transport. Neuroscience.

[86] Hoseinrad H., Shahrestanaki J.K., Moosazadeh Moghaddam M., Mousazadeh A., Yadegari P., Afsharzadeh N. (2021). Protective Effect of Vitamin D3 Against Pb-Induced Neurotoxicity by Regulating the Nrf2 and NF-κB Pathways. Neurotox. Res..

[87] Pierucci F., Garcia-Gil M., Frati A., Bini F., Martinesi M., Vannini E., Mainardi M., Luzzati F., Peretto P., Caleo M. (2017). Vitamin D3 protects against Aβ peptide cytotoxicity in differentiated human neuroblastoma SH- SY5Y cells: A role for S1P1/p38MAPK/ATF4 axis. Neuropharmacology.

[88] Fedotova J., Zarembo D., Dragasek J., Caprnda M., Kruzliak P., Dudnichenko T. (2017). Modulating Effects of Cholecalciferol Treatment on Estrogen Deficiency-Induced Anxiety-Like Behavior of Adult Female Rats. Folia Med..

[89] Libonati L., Onesti E., Gori M.C., Ceccanti M., Cambieri C., Fabbri A., Frasca V., Inghilleri M. (2017). Vitamin D in amyotrophic lateral sclerosis. Funct. Neurol..

[90] Rcom-H’cheo-Gauthier A.N., Meedeniya A.C., Pountney D.L. (2017). Calcipotriol inhibits α-synuclein aggregation in SH-SY5Y neuroblastoma cells by a Calbindin-D28k-dependent mechanism. J. Neurochem..

[91] Li H., Jang W., Kim H.J., Jo K.D., Lee M.K., Song S.H., Yang H.O. (2015). Biochemical protective effect of 1,25-dihydroxyvitamin D3 through autophagy induction in the MPTP mouse model of Parkinson’s disease. Neuroreport..

[92] Calvello R., Cianciulli A., Nicolardi G., De Nuccio F., Giannotti L., Salvatore R., Porro C., Trotta T., Panaro M.A., Lofrumento D.D. (2017). Vitamin D Treatment Attenuates Neuroinflammation and Dopaminergic Neurodegeneration in an Animal Model of Parkinson’s Disease, Shifting M1 to M2 Microglia Responses. J. Neuroimmune Pharmacol..

[93] Lima L.A.R., Lopes M.J.P., Costa R.O., Lima F.A.V., Neves K.R.T., Calou I.B.F., Andrade G.M., Viana G.S.B. (2018). Vitamin D protects dopaminergic neurons against neuroinflammation and oxidative stress in hemiparkinsonian rats. J. Neuroinflamm..

[94] Pettersen J.A. (2017). Does high dose vitamin D supplementation enhance cognition?: A randomized trial in healthy adults. Exp. Gerontol..

[95] Jia J., Hu J., Huo X., Miao R., Zhang Y., Ma F. (2019). Effects of vitamin D supplementation on cognitive function and blood Aβ-related biomarkers in older adults with Alzheimer’s disease: A randomised, double-blind, placebo-controlled trial. J. Neurol. Neurosurg. Psychiatry..

[96] Suzuki M., Yoshioka M., Hashimoto M., Murakami M., Noya M., Takahashi D., Urashima M. (2013). Randomized, double-blind, placebo-controlled trial of vitamin D supplementation in Parkinson disease. Am. J. Clin. Nutr..

[97] Karam C., Barrett M.J., Imperato T., MacGowan D.J.L., Scelsa S. (2013). Vitamin D deficiency and its supplementation in patients with amyotrophic lateral sclerosis. J. Clin. Neurosci. Off. J. Neurosurg. Soc. Australas..

[98] Trojsi F., Siciliano M., Passaniti C., Bisecco A., Russo A., Lavorgna L., Esposito S., Ricciardi D., Monsurro M.R., Tedeschi G. (2020). Vitamin D supplementation has no effects on progression of motor dysfunction in amyotrophic lateral sclerosis (ALS). Eur. J. Clin. Nutrition..

[99] Ghaderi A., Banafshe H.R., Mirhosseini N., Moradi M., Karimi M.-A., Mehrzad F., Bahmani F., Asemi Z. (2019). Clinical and metabolic response to vitamin D plus probiotic in schizophrenia patients. BMC Psychiatry.

[100] Fond G., Godin O., Schürhoff F., Berna F., Bulzacka E., Andrianarisoa M., Brunel L., Aouizerate B., Capdevielle D., Chereau I. (2018). Hypovitaminosis D is associated with depression and anxiety in schizophrenia: Results from the national FACE-SZ cohort. Psychiatry Res..

[101] Thakurathi N., Stock S., Oppenheim C.E., Borba C.P., Vincenzi B., Seidman L.J. (2013). Open-label pilot study on vitamin D_3_ supplementation for antipsychotic-associated metabolic anomalies. Int. Clin. Psychopharmacol..

[102] SanMartin C.D., Henriquez M., Chacon C., Ponce D.P., Salech F., Rogers N.K., Behrens M.I. (2018). *Vitamin D Increases Aβ*140 Plasma Levels and Protects Lymphocytes from Oxidative Death in Mild Cognitive Impairment Patients. Curr. Alzheimer Res..

[103] Owusu J.E., Islam S., Katumuluwa S.S., Stolberg A.R., Usera G.L., Anwarullah A.A., Shieh A., Dhaliwal R., Ragolia L., Mikhail M.B. (2019). Cognition and Vitamin D in Older African-American Women- Physical performance and Osteoporosis prevention with vitamin D in older African Americans Trial and Dementia. J. Am. Geriatr. Soc..

[104] Hiller A.L., Murchison C.F., Lobb B.M., O’Connor S., O’Connor M., Quinn J.F. (2018). A randomized, controlled pilot study of the effects of vitamin D supplementation on balance in Parkinson’s disease: Does age matter?. PLoS ONE.

[105] Zhu D., Liu G.Y., Lv Z., Wen S.R., Bi S., Wang W.Z. (2014). Inverse associations of outdoor activity and vitamin D intake with the risk of Parkinson’s disease. J. Zhejiang Univ. Sci. B.

[106] Appel L.J., Michos E.D., Mitchell C.M., Blackford A.L., Sternberg A.L., Miller E.R., Juraschek S.P., Schrack J.A., Szanton S.L., Charleston J. (2021). The Effects of Four Doses of Vitamin D Supplements on Falls in Older Adults: A Response-Adaptive, Randomized Clinical Trial. Ann. Intern. Med..

[107] Krivoy A., Onn R., Vilner Y., Hochman E., Weizman S., Paz A., Hess S., Sagy R., Kimhi-Nesher S., Kalter E. (2017). Vitamin D Supplementation in Chronic Schizophrenia Patients Treated with Clozapine: A Randomized, Double-Blind, Placebo-controlled Clinical Trial. EBioMedicine.

[108] Gaughran F., Stringer D., Wojewodka G., Landau S., Smith S., Gardner-Sood P., Taylor D., Jordan H., Whiskey E., Krivoy A. (2021). Effect of Vitamin D Supplementation on Outcomes in People with Early Psychosis: The DFEND Randomized Clinical Trial. JAMA Netw. Open..

[109] Gaughran F., Stringer D., Berk M., Smith S., Taylor D., Whiskey E., Landau S., Murray R., McGuire P., Gardner-Sood P. (2020). Vitamin D supplementation compared to placebo in people with First Episode psychosis—Neuroprotection Design (DFEND): A protocol for a randomised, double-blind, placebo-controlled, parallel-group trial. Trials.

